# Preventing Blindness

**Published:** 1892-08

**Authors:** 


PREVENTING BLINDNESS.—An Act for the prevention of blindness has just been made a law by the Rhode Island Legislature. It is similar to the New York State law, and Rhode Island is the first New England State to adopt it. The important paragraph is the first, and it reads as follows:

"Should any midwife or nurse, or person acting as nurse, having charge of an infant in this State, notice that one or both eyes of such infant are inflammed or reddened at any time within two weeks after its birth, it shall be the duty of such midwife or nurse, or person acting as nurse, so having charge of such infant, to report the fact in writing within six hours to the health officer, or some qualified practitioner of medicine, of the city or town in which the parents of the infant reside."

It has been called the "Sore-Eyes Bill." But as the sore eyes in question cause ten per cent. of the cases of blindness, and are the largest single factor, they naturally deserve special, if not legislative, attention.—*New York Medical Record.*

